# Assessing the hodgepodge of non-mapped reads in bacterial transcriptomes: real or artifactual RNA chimeras?

**DOI:** 10.1186/1471-2164-15-633

**Published:** 2014-07-29

**Authors:** Verónica Lloréns-Rico, Luis Serrano, Maria Lluch-Senar

**Affiliations:** EMBL/CRG Systems Biology Research Unit, Centre for Genomic Regulation (CRG), Dr. Aiguader 88, 08003 Barcelona, Spain; Universitat Pompeu Fabra (UPF), Dr. Aiguader 88, 08003 Barcelona, Spain; Institució Catalana de Recerca i Estudis Avançats (ICREA), Pg. Lluis Companys 23, 08010 Barcelona, Spain

**Keywords:** Chimeric RNAs, Fusion transcripts, RNA-seq, Library preparation protocols

## Abstract

**Background:**

RNA sequencing methods have already altered our view of the extent and complexity of bacterial and eukaryotic transcriptomes, revealing rare transcript isoforms (circular RNAs, RNA chimeras) that could play an important role in their biology.

**Results:**

We performed an analysis of chimera formation by four different computational approaches, including a custom designed pipeline, to study the transcriptomes of *M. pneumoniae* and *P. aeruginosa*, as well as mixtures of both. We found that rare transcript isoforms detected by conventional pipelines of analysis could be artifacts of the experimental procedure used in the library preparation, and that they are protocol-dependent.

**Conclusion:**

By using a customized pipeline we show that optimal library preparation protocol and the pipeline to analyze the results are crucial to identify real chimeric RNAs.

**Electronic supplementary material:**

The online version of this article (doi:10.1186/1471-2164-15-633) contains supplementary material, which is available to authorized users.

## Background

In recent years, different groups have shown the existence of RNA editing in different species [1–3], and more recently the possibility of the presence of the so-called RNA chimeras [4, 5]. These are transcripts that contain fragments from independent RNA molecules. RNA chimeras may arise from chromosome translocations, as found in eukaryotes, where they often play a role as transforming oncogenes [6–8]. Furthermore, mRNA chimeras can also arise as product of transcriptional or post-transcriptional events. Examples in the archaeal domain are tRNAs that are composed of pieces transcribed from different regions of the genome [9–12]. Also in eukaryotes, several reports have suggested the presence of RNA chimeras that are not the result of chromosome rearrangement in eukaryotes [13, 14]. This implies the existence of mechanisms for end joining of independent RNA transcripts and, if true, could point out to new hidden complexity. Spliceosome-mediated trans-splicing has been suggested to be one possible mechanism for RNA chimera formation in eukaryotes [13]. Another proposed mechanism for the formation of fusion transcripts is intergenic splicing. This occurs when transcription fails to terminate at a transcription termination site and therefore the nascent transcript is extended until the termination site of the next gene. The resulting bicistronic transcript is processed to obtain a mature RNA formed by exons from two adjacent genes, and these chimeras are often regarded as readthrough chimeras or transcription-induced chimeras [15, 16]. Some recent studies have shown that these chimeras that do not result from chromosomal translocations are translated and may encode for novel functions inside the cell [17, 18].

Several computational approaches have been developed for the detection of chimeric transcripts in RNA-seq data [19–26]. Although all of these computational methods show good sensitivity, true identification of chimeric transcripts could be hampered by experimental artifacts. Indeed, there are reports that indicate that artifactual chimeras could be generated in the RNA sample preparation for deep-sequencing [27, 28]. Multiple factors, including pairwise sequence identity between rRNA genes, number of PCR cycles, and relative abundance of gene-specific PCR templates have been shown to influence chimera formation [27]. Furthermore, the rapid changes in these cutting-edge technologies demand fast adaptation, limiting the time for proper optimization of library preparation protocols, as well as of the software tools used to analyze the results. Thus, it is important to carefully select the mapping software according to the structure of the RNA-seq data under analysis. Some algorithms, as FusionMap [20], ChimeraScan [25] and TopHat Fusion [26] apply different filters in order to validate real chimeric RNAs. For instance, TopHat Fusion [26] considers the total number of reads that cover a given fusion transcript and the read distribution around the chimeric junction, as well as the number of unique reads spanning each junction. It also applies different filters for RNA polymerase read-through (given leaky termination sites, the RNA polymerase would be able to continue transcribing neighbor genes) and minimum anchor length at each side of the junction. ChimeraScan [25] introduces other filters such as the PE mates distance regarding the junction site, and the isoform fraction of the chimera respect to the genes that are forming it. FusionMap [20] also considers read quality in order to discard artifactual chimeras arising from read mismapping.

Here, we want to determine if prokaryotic RNA chimeras exist and to what extent they may be the result of artifact generation both in the library preparation protocols and the analysis methods. To do so we chose two bacteria belonging to gram negative (*Pseudomonas aeruginosa*) and gram positive (*Mycoplasma pneumoniae*) classes. Both genomes are fully sequenced [29, 30] and in the case of *M. pneumoniae,* aside from being very small (816 Kb) a genome-wide transcriptome analysis is available [31]. To control for potential artifacts in sample preparation we mixed *P. aeruginosa* and *M. pneumoniae* at different stages of the RNA extraction and preparation, and performed RNA-seq of the isolated and mixed samples. We analyzed two widely used protocols for library preparation (Directional RNA-seq (TruSeq small RNA Sample Prep Kit, Illumina) and TruSeq stranded RNAseq (TruSeq Stranded mRNA Sample Prep Kit, Illumina), see Methods) to study the impact of these protocols on chimera formation. Both protocols are widely used in the analysis of eukaryotic and prokaryotic transcriptomes. Whilst the first one is mainly used for small RNAs in eukaryotes, such as siRNAs or microRNAs, the second is mostly used for mRNA sequencing [32–35]. Regarding bacteria, both methods have been used for total RNA library preparation [36, 37]. To analyze the data, we used FusionMap [20], ChimeraScan [25] and TopHat-Fusion [26] (see Additional file 1: Figure S1) as well as a customized pipeline designed to filter out artifacts.

Our analysis detected large numbers of putative chimeric RNAs that were revealed to be artifacts generated during library preparation protocols, using widely accepted sequencing methodologies. Also, we found that the methodology used for sequence analysis could result in different non-real chimeras identified. Therefore, care should be taken with the selection of a sequencing protocol and software for the analysis when annotating fusion transcripts in different organisms.

## Results and discussion

### Sequence mapping

**Figure 1:**
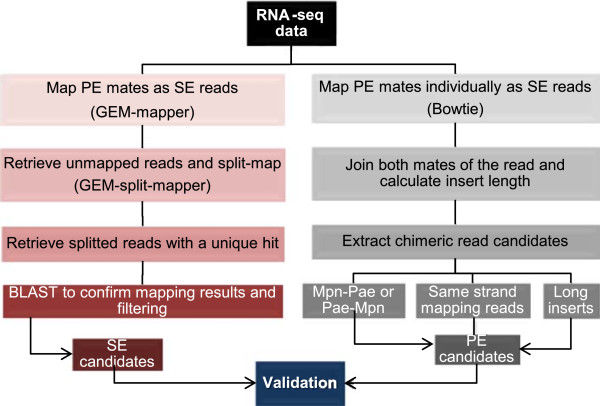
**Pipeline of the RNAseq data analysis.** Two complementary approaches have been integrated to analyze the putative chimeric RNAs: i) mapping pair end mates as single end by using GEM-mapper and ii) mapping pair ends individually as single ends using Bowtie. After the two complementary mapping strategies there is a validation procedure that integrates three steps: i) determination of confidential scores that consider the levels of expression of the genes that originate the chimeric RNA; ii) filter by unique reads that considers the number of different reads that represent the chimeric junction and iii) filter by staircase profile that select for those chimeric variants that show a homogenous distribution of counts for the different reads that cover the junction.

To analyze the origin of chimeric RNAs and to discern if they are artifacts derived of library preparation or natural chimeric RNAs, we prepared different samples by mixing RNA of *M. pneumoniae* and *P. aeruginosa* at different ratios (1:1; 1:5 and 1:50), as well as non-mixed samples. Mixing was done immediately after RNA extraction. Another sample was obtained by mixing the cells of both bacteria prior to RNA extraction (Cell Mix). Libraries were prepared using the Directional RNA-seq library protocol (see Methods). We used two alternative short read mappers: GEM-mapper and Bowtie [38, 39]. With GEM-mapper the sequences were mapped individually as single-end (SE) reads without considering the associated mates, while Bowtie was used to map paired-end (PE) mates and calculate the insert lengths in between both mates (Figure 1). In both cases it was requested that the reads mapped uniquely to a single position in the genome. It has been previously reported that in PE sequencing a small percentage of second mate reads are not correctly assigned to their corresponding first mate read. This will lead to the obtaining of artifactual chimeras [40]. In contrast to the previous methods, we used SE reads in the first step of the analysis to identify the junction of putative chimeric RNAs. Then, we applied PE mapping to validate the candidates for the study of putative chimeric RNAs in all the samples previously described, by supporting the reads that spanned the chimeric RNA junction. Both SE and PE reads were used to validate the chimeric candidates, and several filtering steps were applied in order to discard potential artifacts arising from the different steps of library preparation protocols.

Deep sequencing analysis of *M. pneumoniae* and *P. aeruginosa* samples in the RNA mix samples showed a significant number of chimeric candidates (33389 in the 1:1 ratio RNA mix sample), the majority of them involving tRNAs and rRNAs in both species (for instance, in the 1:1 ratio RNA mix sample, 70.6% of the chimeras were formed by two rRNAs, and 0.96% were formed by two tRNAs, see Additional file 1: Figure S1), as well as a significant proportion of circular RNAs (10.8% of the rare isoforms observed in the 1:1 ratio RNA mix sample corresponded to putative circular RNAs, 99.3% of them were formed in rRNAs, the remaining 0.7% correspond to circular RNAs formed by tRNAs; Additional file 1: Figure S1). We also identified a small percentage (0.02%) of chimeric mRNAs. However, we found that the second ORF was kept in frame in around 33% of the mRNA chimeras, as expected by chance, for inter- and intra-species mRNA chimeras, thus indicating random fusion events.

**Figure 2:**
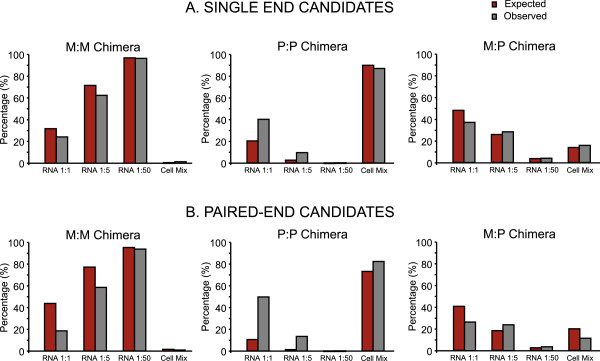
**Identification of putative chimeric RNAs. A)** Histograms show, from left to right, the percentage of different chimeric RNAs (M:M = *M. pneumoniae*:*M. pneumoniae*; P:P = *P. aeruginosa*:*P. aeruginosa*; M:P = *M. pneumoniae*:*P. aeruginosa*) obtained after single end RNAseq data analysis of the samples obtained by mixing RNAs in the different ratios (1:1; 1:5; 1:50 and Cell mix). Grey columns indicate the percentage of expected chimeras calculated by considering the total number of reads of each species per sample and red columns indicated the percentage of obtained chimeric RNAs after RNAseq data analysis **B)** Histograms obtained after analyzing RNA-seq data by pair end. The data is represented following the same criteria than described for panel **A**.

In the RNA mix experiment at a ratio 1:1 we found around 30% of *M. pneumoniae* chimeras, 50% of *P. aeruginosa* and 20% with RNAs from both species (Figure 2). Assuming no biases towards the formation of chimeras intra- and inter-species, we calculated the expected percentage of chimeras of each class in the experiments corresponding to the different mixtures of RNA. In this regard, it is worth considering that while *M. pneumoniae* has one rRNA operon and 32 tRNAs, *P. aeruginosa* has four rRNA operons and 63 tRNAs. For this purpose we considered the total number of fully mapped reads of each bacterium per sample. Data analysis showed that the percentage of chimeras in which the two parts corresponded to only *M. pneumoniae,* or the two species was similar to what was expected by chance (Figure 2). In the case of *P. aeruginosa* there could be a higher proportion of observed chimeras than expected. In the Cell Mix sample, there were initially many more *P. aeruginosa* cells than *M. pneumoniae* cells, based on the number of reads that fully mapped each species. Thus, both the expected and observed values for the chimeras of *P. aeruginosa* are much higher than in the RNA mix samples, in which the proportion of RNA was favoring *M. pneumoniae* (Figure 2).

All these results indicate that most chimeras observed are artifacts of the library preparation. For instance, a phenomenon named ‘template switching’ , consisting in an exchange of templates of the reverse transcriptase, has been reported to occur whenever duplicated sequences are close to the junction site [41].

### Filtering criteria to discern in between real versus artifactual chimeric RNAs

The initial analysis of the RNA-seq data described above revealed the need to create an objective criterion to discern between artifactual or real chimeric RNAs.

First, different filters were applied to the preliminary sets of SE and PE candidates to discard the potential artifacts previously reported [41]. For example, to remove the ‘template switching’ effect [41], all the SE candidates that presented duplicated sequences of more than 6 nucleotides at both sides of the junction were discarded as likely reverse transcription artifacts during the process of library preparation. Then, we grouped all SE candidates spanning the exact same junction site. In fact, a vast majority of chimeric candidates (around 80%) was supported by only one read and were thus discarded.

**Figure 3:**
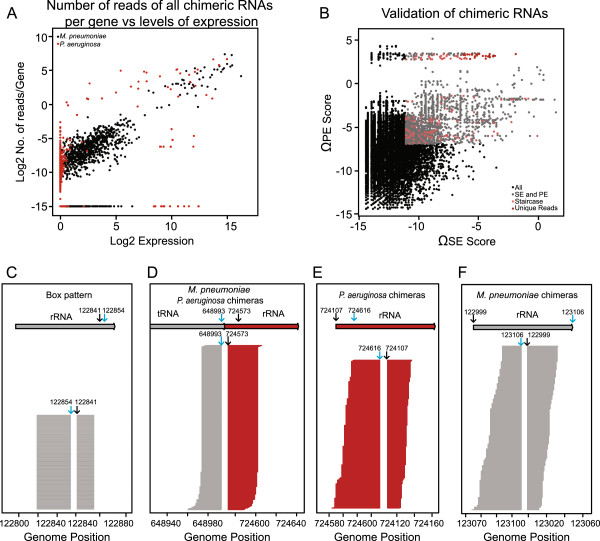
**Validation of putative chimeric RNAs. A)** *Relation between levels of expression and number of chimeras.* The graph represents the number of chimeric reads per gene versus gene expression levels for *M. pneumoniae* (in black) and *P. aeruginosa* (in red). Sample obtained after mixing RNA of *M. pneumoniae* and *P. aeruginosa* in a ratio 1:1. **B)** *Representation of putative chimeric RNAs in M. pneumoniae after validation filters.* Black dots represent the 43.399 initial candidates. Grey dots, putative chimeric RNAs after filtering by Ω_se_ and Ω_pe_ scores (4047). Pink dots, candidates after applying staircase filter (74). Dark red dots,the putative chimerias obtained after applying the unique reads filter (33). **C)** Representation of a “box-pattern” profile of the reads corresponding to a *M. pneumoniae* chimeria comprising two regions of the rRNA. Grey arrow represents the genomic regions of *M. pneumoniae* and blue and black arrows, the junction positions in the pileup of the sequenced reads. **D)** *Representation of reads for an artificial chimeric RNA result of ligation of tRNA of M. pneumoniae and rRNA of P. areuginosa.* Arrows represent the genomic regions of *M. pneumoniae* (in grey) and *P. aeruginosa* (in dark red). Blue and black arrows indicate the positions of the junction in the genome and in the pileup of the sequenced reads. **E)** *Staircase profile can be observed for the different reads that cover the junction of the chimeric RNA in P. aeruginosa.* Dark red arrow represents the genome region that codes for the RNA. Blue and black arrows indicate the positions of the junction in the genome. **F)** *Representation of reads for a putative chimeric RNA in M. pneumoniae.* Grey arrow represents the genome region that codes for the RNA. Blue and black arrows, the junction positions in the pileup of sequenced reads.

Second, we considered the expression levels of the genes located at both sides of the junction. The expression values were calculated based on the fully mapped reads from the RNAseq datasets. The reason for considering expression levels is the fact that in *M. pneumoniae*, there is a good correlation between the expression of a gene and the number of chimeric fragments that map inside that gene (Figure 3A) (r = 0.694). This correlation is much lower in *P. aeruginosa* (r = 0.267). The reason behind the poor correlation in *P. aeruginosa*, is the high abundance of ribosomal RNA in the samples (93.3% in *P. aeruginosa*, and 81.2% in *M. pneumoniae*), which results in lower deep sequencing coverage (we obtained roughly the same number of sequencing reads for *M. pneumoniae* and *P. aeruginosa*, but the latter has a much larger genome and four rRNA operons). Therefore, to find a true chimera, the number of reads supporting the junction has to be significant compared to the expression of the genes forming the chimera. This issue was assessed via two confidence scores (Ω_se_ and Ω_pe_, SE score and PE score; see Methods section). These scores relate the number of reads of a chimeric RNA to its expression. The least expressed gene comprising the chimera was chosen to evaluate the expression, as the limiting factor for its formation. Figure 3B represents the distribution of the scores for the 1:1 RNA mixture sample.

Additionally, it has been proposed that the pattern of the reads covering the junction may reveal the existence of possible false positive chimeras arising from PCR artifacts or errors in the mapping process [42, 43]. If all reads covering a junction are identical, i.e. mapping to the exact same positions, and thus they show a box-like pattern when the coverage of the junction is represented (Figure 3C), this chimeric RNA is likely to be an artifact. Contrarily, if the reads spanning the junction are distributed along the chimera and show a staircase-like pattern (Figure 3E and 3F), the chimera can be assumed to be real [42, 43]. We followed two criteria that allowed us to distinguish in between these two patterns. First of all, we considered the number of unique reads (non-duplicated reads) that spanned each junction. We related this number to the total reads that mapped to that junction. Furthermore, we required that the distribution of the reads covering the same junction was uniform and thus showing a staircase pattern (see Methods). This second criteria was added when a mixed pattern was found to occur in the false positive set of chimeras *M. pneumoniae*:*P. aeruginosa*. In this third case a staircase-like shape is also observed, but here, one unique read likely coming from a PCR artifact is responsible for more than 50% of the total observed read counts (Figure 3D). Figure 3B depicts the chimeras passing the different filtering criteria established, representing the Ω_se_ and Ω_pe_ scores.

In order to determine which threshold to use for each of the filters applied, we used the 1:1 RNA mixture sample and we divided the fusion transcript candidates into two groups, one formed by inter-species chimeras (true false positives) and another formed by intra-species chimeras. We tested different sets of parameters and used the ones that were able to eliminate the inter-species chimeras in the final set of results. With this approach, in the end we were able to obtain a set of 29 *P. aeruginosa* fusion transcripts in the 1:1 RNA mixture sample that passed all the thresholds. All of these RNAs represent a circular RNA formed in the 23S rRNA. No chimeric RNAs were found in *M. pneumoniae* in this sample.

**Figure 4:**
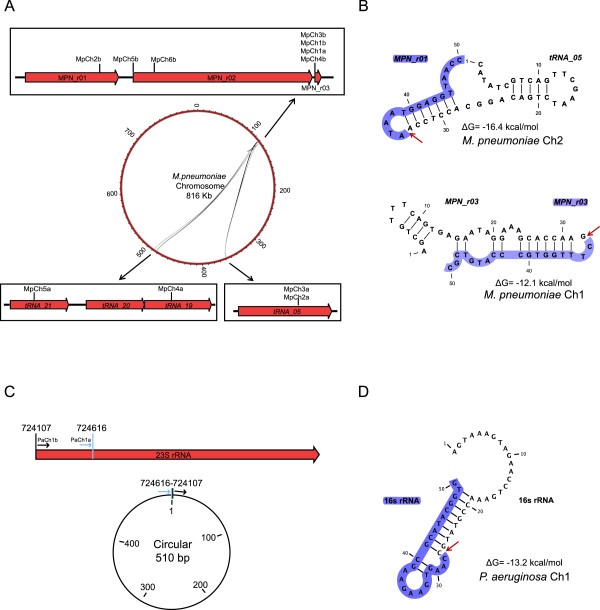
**Putative chimeric RNAs in** ***M. pneumoniae*** **and** ***P. aeruginosa*** **. A)** *Circos plot representing chimeric RNAs in the genome of* *M. pneumoniae.* Rectangles represent a zoom of the genomic region indicated with a black arrow. These regions correspond to the two parts that compound the different chimeric RNAs described in Additional file 2: Table S2 (a = region of first gene and b = region of second gene). Red arrows in the rectangles indicate genes and lines in Circos plot indicate connections between genomic regions. **B)** *Secondary structure of M. pneumoniae RNA species*. Upper part of the panel shows the predicted secondary structure of a putative chimeric RNA of *M. pneumoniae* (MpnCh2) obtained by CLC Workbench program. Lower panel shows an example of the secondary structure for the MpnCh1, a circular RNA of *M. pneumoniae*. In both representations the junction is indicated with a red arrow and second part of the chimeric RNA is shadowed in blue. Complementary regions are connected with black lines. **C)** *Circular RNA of P. aeruginosa*. Circular RNA corresponding to the results described in Additional file 2: Table S3. Red arrow indicates 23S rRNA gene, blue and black lines indicate the genome positions of the first and second regions of the circular molecule, respectively. Circular graph is a schematic representation of the obtained molecule. **D)** *Secondary structure of the 23s rRNA chimera found in P. aeruginosa*. The junction is indicated with a red arrow and second part of the chimeric RNA is shadowed in blue. Complementary regions are connected with black lines.

We applied the filtering with these same thresholds to the remaining samples. It was observed that in the RNA mixtures 1:5 and 1:50, in which the *P. aeruginosa* RNA concentration decreased, no chimeras appeared from this species and only few chimeras of *M. pneumoniae* were found. All putative chimeras found after the filtering are represented in Figure 4.

### Analysis by other software tools

After performing strand specific paired-end RNA-seq [44], sequencing data was analyzed by three different software tools aimed to detect fusion transcripts in eukaryotic RNA-seq data: FusionMap [20], ChimeraScan [25] and TopHat Fusion [26]. All these three pipelines were applied using the specified default parameters except for the maximal intron length. As no introns are present in bacterial transcriptomes, this distance was minimized in the analysis, in order to find chimeras regardless the distance in between the two parts forming them. After applying each of these pipelines to the 1:1 RNA mixture dataset, we observed large discrepancies among the results. The number of chimeras detected by the different pipelines varied largely (see Additional file 2: Table S1). Whereas TopHat Fusion found up to 350 chimeric RNAs in this sample, which were grouped into 51 sets according to their genomic location, ChimeraScan and FusionMap found more than 3000 fusion transcripts each, and ours only 29, but grouped in one unique set according to their genomic location (they only were formed in the same region of the 23S rRNA of *P. aeruginosa*)*.* Furthermore, the three methods interrogated found chimeras formed by transcripts different than rRNAs and tRNAs, the most abundant ones in bacterial genomes. Whereas TopHat Fusion found 16 fusion transcripts formed by protein-coding or non-coding RNAs (4.57% of the total chimeras detected), FusionMap found 32 (0.88%) and ChimeraScan found 22 (0.71%). Interestingly, as shown in the case of the 1:1 RNA mixture of both bacteria, not a single common fusion transcript was found by all three methods (Additional file 3: Figure S2A). Also, all different pipelines (except ours) found inter-species chimeras, providing proof of the presence of false positives in the results (Additional file 3: Figure S2B). These results should be regarded with caution since the three used methods were developed for analysis of eukaryotic and not prokaryotic RNAseq data.

### Avoiding hodgepodge in RNA-seq data

The fact that some chimeras passed all the established thresholds does not imply that they are real RNA chimeras. In fact, no coincidences were found among the different protocols used to analyze the data (Additional file 2: Table S1 and Additional file 3: Figure S2A). Furthermore, chimeras found in *M. pneumoniae* were only derived from rRNAs and tRNAs, the most abundant species. However, they were not found randomly within these molecules, but they appeared to be formed in between certain ‘hotspots’ (Figure 4A). We observed that in many cases, these ‘hotspots’ corresponded to regions that showed base-pair complementarity (Figure 3B). In the case of *P. aeruginosa* the only RNA species that passed all filters using our custom pipeline in the different RNA-seq experiments is a circular form of the 23S rRNA (Figure 4C) and there are 10 bps in both sides of the junction with exact base-pair complementarity (Figure 4D). Therefore, we hypothesized that the strong secondary structure of the native molecules prevents their fully denaturalization during sample preparation using the Directional RNA-seq library preparation method (see Methods, Directional RNA-seq library preparation and sequencing). Thus, after shearing the RNA molecules by sonication, the fragments that kept hybridized could remain close in the space, favoring their ligation in the following step of the library preparation.

**Figure 5:**
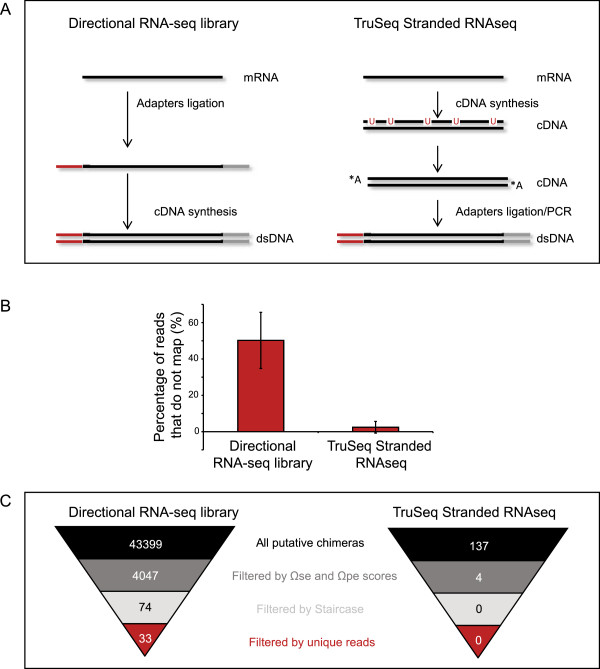
**Comparison between different methodologies to prepare RNA-seq libraries. A)** Schematic representation of the Directional RNA-seq and TruSeq Stranded RNA-seq protocols. **B)** Histogram representing the percentage of reads that do not map against *M. pneumoniae* genome in the different protocols used to prepare RNA-seq libraries. **C)** The inverted pyramids indicate the different number of chimeric RNAs obtained in the consecutive steps of sample validation for both protocols of library preparation.

To test if double-stranded RNA could be behind the observed chimeras we used a different protocol (TruSeq stranded RNA-seq) to prepare the libraries for deep sequencing of the *M. pneumoniae* unmixed RNA sample (Figure 5A). In this protocol, cDNA is synthesized before the adapter ligation, and therefore the secondary structure of these molecules is removed (see Methods). After sequencing the sample prepared with this second protocol, we observed that the number of initially non-mapped reads to the reference genome decreased dramatically (from 50% to 5%; Figure 5B). After performing the analysis and filtering previously described, the number of chimera candidates decreased to 137 (compared to several thousands obtained with the first protocol; see Figure 5C) and no chimeric reads were found in the final filtering steps (Figure 5C). Also, when applying the three different published pipelines stated above, no chimeras were found in any of them (See Additional file 1: Figure S1). All these data taken together suggests that the TruSeq stranded RNA-seq is the most accurate protocol to avoid artifacts in the library preparation and the described pipeline allows the filtering of artifactual chimeric RNAs. Directional RNA-seq is widely used for microRNA library preparation in eukaryotes and RNAseq in prokaryotes, which could lead to identification of non-real chimeras and/or underestimation of the expression levels of some genes.

## Conclusions

Nowadays, RNA-Seq is becoming one of the most used approaches for transcriptome profiling. Different studies using this methodology have suggested the existence of chimeric RNAs and circular rRNAs in different organisms. We showed that these RNAs could be artifacts generated during library preparation, and that they are protocol-dependent. We compared two different protocols for RNA-seq library preparation and observed that theyled to very different results in terms of reported chimeric RNAs. Moreover, different sequence analysis methodologies including ours don’t eliminate all non-real chimeras. Therefore, care should be taken when selecting a protocol for library preparation and sequencing, as well as a pipeline for the analysis of the results.

## Methods

### Bacterial strains and growth conditions

*M. pneumoniae* M129 was grown in 25 cm^2^ tissue culture flasks with 5 mL of modified Hayflick medium at 37°C as previously described [45]. *Pseudomonas aeruginosa* PAO1 cells were grown at 37°C under agitation (100 rpm) in 25 ml of 7H9 broth medium from BD plus ADC with 0.5% glycerol and 0.05% tween80.

### RNA extractions and sample preparations

After growing *M. penumoniae* during 96 h at 37°C, cells were washed twice with PBS and lysated with 700 μl of Qiazol buffer.

*P. aeruginosa* cells were grown during 4 days at 37°C, after cell centrifugation the pellet was washed twice with PBS. Then, samples were lysated with 700 μl of Qiazol buffer.

RNA extractions were performed by using miRNeasy mini Kit (Qiagen) following the instructions of the manufacturer.

RNAs obtained from two bacteria were mixed in different proportions: 1:1, 1:5 and 1:50 (ratio *P. aeruginosa:M. pneumoniae*). These mixtures together with non-mixed wild-type RNAs were used for RNA-seq library preparation.

Also a RNA extraction was performed after mixing the cells 1:1 (*M. pneumoniae*:*P. aeruginosa*).

### Library preparations

Libraries for RNA-seq were prepared following two different protocols: i) Directional RNA-seq library preparation and sequencing; ii) Illumina TruSeq stranded RNAseq sample preparation.

#### Directional RNA-seq library preparation and sequencing

One μg of total RNA was fragmented to around 100–150 nt using NEB Next Magnesium RNA Fragmentation Module (ref. E6150S, NEB). Treatments with Antarctic phosphatase (ref. M0289S, NEB) and PNK (ref. M0201S, NEB) were performed in order to make the 5′ and 3′ ends of the RNA available for adapter ligation. Samples were further processed using the TruSeq small RNA Sample Prep Kit (ref. RS-200-0012, Illumina) according to the manufacturer’s protocol. Briefly, 3′ adapters and subsequently 5′ adapters were ligated to the RNA. cDNA was synthesized using reverse transcriptase (SuperScript II, ref. 18064–014, Invitrogen) and a specific primer (RNA RT Primer) complementary to the 3′ RNA adapter. cDNA was further amplified by PCR using indexed adapters supplied in the kit. Finally, size selection of the libraries pas performed using 6% Novex® TBE Gels (ref. EC6265BOX, Life Technologies). Fragments with insert sizes of 100 to 130 bp were cut from the gel, and cDNA was precipitated and eluted in 10 μl EB.

#### Illumina TruSeq stranded RNAseq sample preparations

Libraries were prepared using the TruSeq Stranded mRNA Sample Prep Kit v2 (ref. RS-122-2101/2) according to the manufacturer’s protocol. Briefly, 1 μg of total RNA were fragmented to approximately 300 bp. cDNA was synthesized using reverse transcriptase (SuperScript II, ref. 18064–014, Invitrogen) and random primers. The second strand of the cDNA was done by removing the RNA template and synthesizes a replacement strand, incorporating dUTP in place of dTTP to generate ds cDNA. Then ds cDNA was used for library preparation. dsDNA was subjected to end repair, addition of “A” bases to 3′ ends and ligation of the barcoded Truseq adapters. All purification steps were performed using Qiagen PCR purification columns (refs. 50928106 and 50928006). Library size selection was done with 2% low-range agarose gels. Fragments with insert sizes of 200 to 400 bp were cut from the gel, and DNA was extracted using QIAquick Gel extraction kit (ref. 50928706, Qiagen) and eluted in 20 μl EB. Library amplification was performed by PCR on the size selected fragments using the primer cocktail supplied in the kit.

Final libraries were analyzed using Agilent DNA 1000 chip to estimate the quantity and check size distribution, and were then quantified by qPCR using the KAPA Library Quantification Kit (ref. KK4835, KapaBiosystems) prior to amplification with Illumina’s cBot. Libraries were loaded at a concentration of 1.66 pM onto the flowcell, and were paired-end sequenced, with a mate read length of 50 nts on Illumina’s HiSeq 2000.

### RNA-seq data analysis with a custom-designed pipeline

The workflow followed to identify the chimeric RNA candidates is shown in Figure 2. This pipeline can be divided in three sections: first, we identified the putative chimeric junctions by split-mapping the single mates of the paired-end reads. Then, the information of the paired-end reads was used to confirm the existing junctions and determine the putative chimeras. Finally, the putative chimeras were validated by using three filters: i) Confidence scores; ii) Filtering by unique reads and iii) Filtering by a staircase-like profile.

#### Split-mapping

For each experiment, both mates of the paired-end reads were considered as independent single-end reads. They were mapped using GEM [39] to the reference genomes of *M. pneumoniae* M129 or *P. aeruginosa* PAO1. For the experiments in which both RNAs were mixed, reads were mapped against an artificial genome formed by the union of the chromosomes of both species.

As no rRNA depletion was performed during sample preparation, the vast majority of the mapped reads correspond to the rRNAs of both bacteria. *P. aeruginosa* has four rRNA operons. Three of them were removed artificially from the reference genome, considering the remaining one as the reference rRNA operon. This was aimed not to discard all putative chimeras coming from any of them.

Reads that were not mapped in this first round were retrieved and remapped using GEM-split-mapper, a component of the GEM suite [39]. This mapper splits the input reads in two parts and tries to map both of them to different sites of the genome. Again, up to two mismatches were allowed. After this second mapping, only those reads that yielded one unique hit in the reference genome (this is, the reads that uniquely split-map to two different locations) were considered for subsequent analysis.

In order to validate the mapping of the split reads, a BLAST search of them against the reference genome was performed. Only the reads showing an agreement in between the split-mapping and the first hits of the BLAST results were retrieved and considered as single-end candidates (SE candidates; Figure 2).

#### Paired-end mapping

Both mates of the paired-end reads were mapped separately as single-end reads using Bowtie [38]. In this case, a full mapping of each mate was expected, with up to two mismatches. Mates were mapped to any of the reference genomes aforementioned, according to the RNA composition of each sample. After mapping both mates, they were joined to obtain the paired-end information and calculate the insert length. The insert length distribution obtained was used to determine which paired-end reads could be considered to have an abnormal insert size and thus be chimeric RNA candidates.

In addition to the reads presenting an abnormal insert length, paired-end reads in which each of the mates were mapping to different species were extracted, knowing that this set of chimeric reads will only correspond to false positive hits. Paired-end reads in which both ends were mapping to the same strand were also retrieved, as well as reads in which the second mate was mapping upstream the first mate, being candidates to represent putative circular RNAs.

### Candidate filtering and validation

#### Determination of confidence score for chimeric RNAs

To discern between real and artifactual chimeric RNAs we have defined two scores (Ω_se_ and Ω_pe_) based on the two different methods for the analysis (single-end, se; paired-end, pe). Each of them considers the number of reads supporting each chimeric RNA and the gene expression levels of the different mRNAs that comprise this hybrid RNA. The scores are calculated as follows:


Ω_se_ and Ω_pe_ are the confidence scores for single-end and paired-end analysis, respectively; Nc_se_ and Nc_pe_ are the total number of reads that define a chimeric RNA in the different approaches performed; N_t_ is the total number of reads that map canonically to the gene with the lowest expression value of the chimera (which will be limiting in the chimera formation); L is the gene length of the least expressed gene in the chimera.

#### Filtering by unique reads and staircase-like profile

Here, we considered the number of unique reads (non-duplicated reads) spanning each chimeric junction. To establish a threshold, we used a score that related the number of unique sequences per chimera to the number of total reads mapping to this same chimera.

In addition to that, we also considered the distribution of the reads spanning the chimeric junction. This distribution should be uniform and there should not be any over-represented read, showing a staircase pattern upon coverage representation (Figure 3E, 3F). To establish a threshold for this criterion, we determined the percentage of reads corresponding the most abundant unique sequence respect to the total of reads spanning the junction.

## Electronic supplementary material

Additional file 1: Figure S1: Percentages of different RNA molecules. Histogram represents the percentages of different chimeric RNAs in the 1:1 ratio RNA mix sample (rRNA = ribosomal RNA; tRNA = transfer RNA; mRNA = RNA of ORFs and non-coding RNAs). Pie chart represents the percentage of tRNA and rRNA that are circular or chimeric RNAs. Values next to the pie charts for the rRNA-rRNA and tRNA-tRNA bars represent the percentage of circular RNAs from the total of chimeras of these classes. (PDF 289 KB)

Additional file 2: Table S1: Putative chimeras found by the 4 pipelines tested in two samples from different library preparation protocols. Numbers in parenthesis represent the number of chimeras clustered by genomic location (if applicable). **Table S2.** *Mycoplasma pneumoniae* chimeric RNAs obtained after validation in directional RNA-seq library preparation. **Table S3.** *Pseudomonas aeruginosa* chimeric RNAs obtained after validation in directional RNA-seq library preparation. **Table S4.** Putative chimeric RNAs in the 1:1 RNA mixture found by TopHat-Fusion. **Table S5.** Putative chimeric RNAs in the 1:1 RNA mixture found by FusionMap. **Table S6.** Putative chimeric RNAs in the 1:1 RNA mixture found by ChimeraScan. (XLSX 1 MB)

Additional file 3: Figure S2: Results of the different pipelines used to analyze the data. A) Venn diagram showing the number of chimeras found by each of the pipelines used and the concordances among them. The diagram shows the values for the analysis of the 1:1 RNA mix sample, using the directional RNA-seq library preparation protocol. B) Bar plot showing the percentages of chimeras of each type found by the different pipelines tested. THF: TopHat-Fusion, CS: ChimeraScan, FM: FusionMap, OURS: Our custom designed pipeline. (PDF 458 KB)

## Abbreviations

PE: Paired-end
SE: Single-end
THF: TopHat-Fusion
CS: ChimeraScan
FM: FusionMap
Ω_se_: Single-end score
Ω_pe_: Paired-end score.
