# Supplementary figures and images for: Assessing the hodgepodge of non-mapped reads in bacterial transcriptomes: real or artifactual RNA chimeras?
Source: BMC Genomics. 2014 Jul 29;15(1):633. doi: 10.1186/1471-2164-15-633 (PMC4122791; doi:10.1186/1471-2164-15-633)

Figure S1

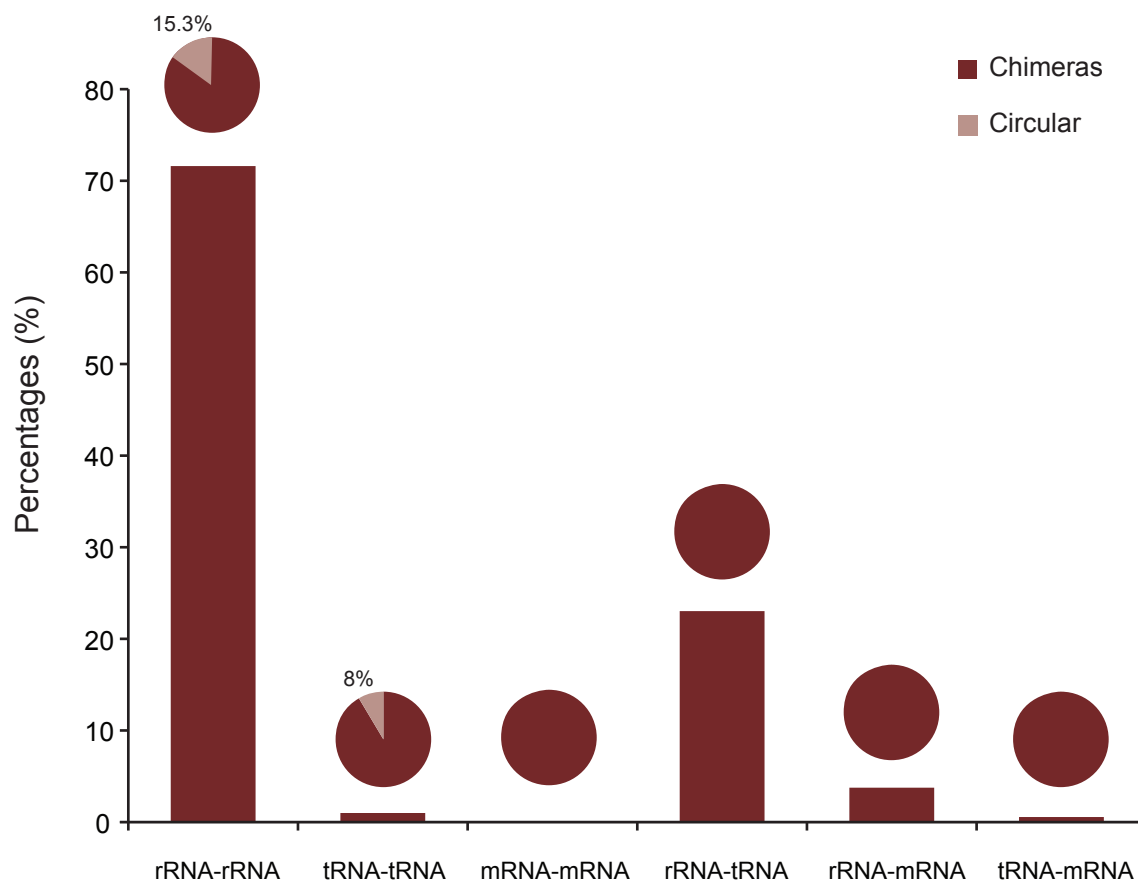

Supplement: Supplementary file 1 — Additional file 1: Figure S1: Percentages of different RNA molecules. Histogram represents the percentages of different chimeric RNAs in the 1:1 ratio RNA mix sample (rRNA = ribosomal RNA; tRNA = transfer RNA; mRNA = RNA of ORFs and non-coding RNAs). Pie chart represents the percentage of tRNA and rRNA that are circular or chimeric RNAs. Values next to the pie charts for the rRNA-rRNA and tRNA-tRNA bars represent the percentage of circular RNAs from the total of chimeras of these classes. (PDF 289 KB) [file 12864_2014_6315_MOESM1_ESM.pdf]

### Figure S2

A

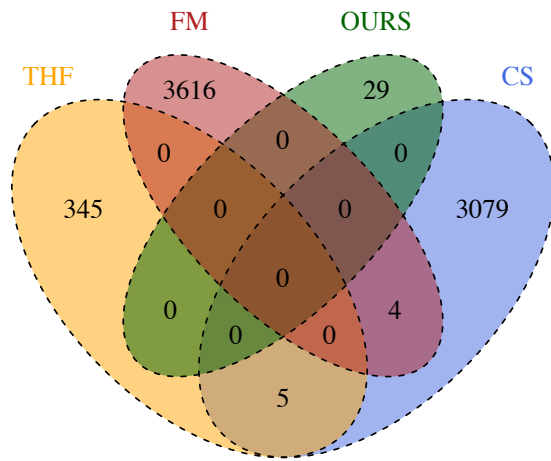

B

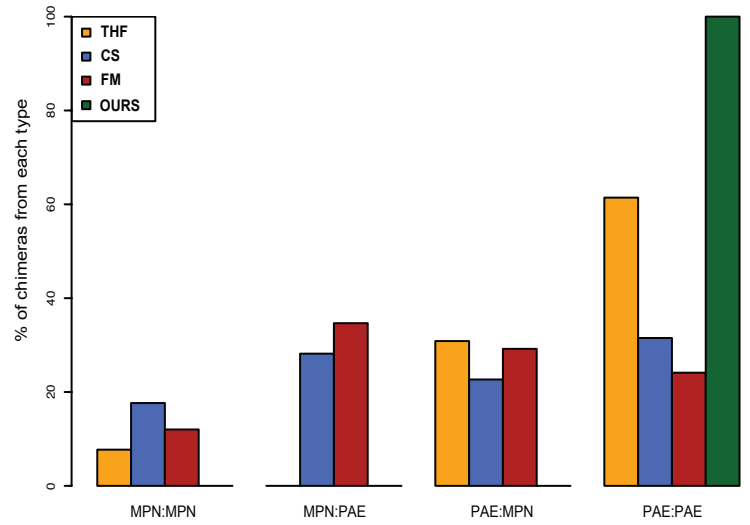

Supplement: Supplementary file 3 — Additional file 3: Figure S2: Results of the different pipelines used to analyze the data. A) Venn diagram showing the number of chimeras found by each of the pipelines used and the concordances among them. The diagram shows the values for the analysis of the 1:1 RNA mix sample, using the directional RNA-seq library preparation protocol. B) Bar plot showing the percentages of chimeras of each type found by the different pipelines tested. THF: TopHat-Fusion, CS: ChimeraScan, FM: FusionMap, OURS: Our custom designed pipeline. (PDF 458 KB) [file 12864_2014_6315_MOESM3_ESM.pdf]
